# Tumor Cell Seeding During Surgery—Possible Contribution to Metastasis Formations

**DOI:** 10.3390/cancers3022540

**Published:** 2011-06-08

**Authors:** Pachmann Katharina

**Affiliations:** Department of Experimental Hematology and Oncology, Clinic for Internal Medicine II, Friedrich Schiller University, Jena D-07747, Germany; E-Mail: katharina.pachmann@med.uni-jena.de

**Keywords:** surgery, tumor cell dissemination, monitoring of circulating epithelial tumor cells

## Abstract

In spite of optimal local control in breast cancer, distant metastases can develop as a systemic part of this disease. Surgery is suspected to contribute to metastasis formation activating dormant tumor cells. Here we add data that seeding of cells during surgery may add to the risk of metastasis formation. The change in circulating epithelial tumor cells (CETC) was monitored in 66 breast cancer patients operated on with breast conserving surgery or mastectomy and during the further course of the disease, analyzing CETC from unseparated white blood cells stained with FITC-anti-EpCAM. An increase in cell numbers lasting until the start of chemotherapy was observed in about one third of patients. It was more preeminent in patients with low numbers of CETC before surgery and, surprisingly, in patients without involved lymph nodes. Patients with the previously reported behavior—Reincrease in cell numbers during adjuvant chemotherapy and subsequent further increase during maintenance therapy—were at increased risk of relapse. In addition to tumor cells already released during growth of the tumor, cell seeding during surgery may contribute to the early peak of relapses observed after removal of the primary tumor and chemotherapy may only marginally postpone relapse in patients with aggressively growing tumors.

## 1. Introduction

In breast cancer it is rarely the primary tumor, but rather the metastases that determine the fate of the patient. Such metastases grow from cells seeded from the tumor into the circulation. In only 10% of cases is the tumor primarily metastatic; in most patients metastases develop after the primary tumor has been removed by surgery.

This led to the assumption that metastases may arise from cells released from remnant tumor tissue left in the breast after surgery. According to the Halsted doctrine [1], therefore, the aim of surgery was to remove as much tissue as possible leading to a radical and often disfiguring mastectomy. Despite these radical procedures, however, relapses still occurred.

Therefore, it was concluded that the tumors must have a systemic part which cannot be eradicated even by the most radical surgery. Less radical approaches resulting frequently in breast-conserving surgery were shown to improve cosmetic results with no inferiority in disease-free and overall survival [2].

If breast cancer is regarded as a systemic disease, this implies that tumor cells can already leave the tumor during tumor growth. It seems that increasingly more cells are seeded during tumor growth [3], increasing the chance that one of these cells will settle in an appropriate niche and develop into a metastasis. Tumor cell-platelet interactions could enable dissemination by forming cell aggregates that protect tumor cells by collaborating during extravasation [4]. In some cases, metastases appear in distant organs even after a decade of relapse-free survival.

Therefore, the most promising approach would be to detect and remove the primary tumor before it has grown large enough to seed cells.

It has, however, long been suspected that surgery itself can lead to increased dissemination of tumor cells [5-7] which lead to the “no touch” recommendation by Turnbull *et al.* [8] in colon cancer surgery. We, too, could show that epithelial cells are released during breast surgery [9]. This release was not an immediate event. A wave of cell dissemination was seen only 3–7 days after surgery. This may be due to the fact that during surgery cells presumably are forced into the lymphatics and subsequently drained into blood [10].

We showed that in some of the patients, the numbers of circulating epithelial cells (CETC) returned to pre-surgery values but in other patients CETC numbers remained at an elevated level for extended periods after surgery. Cell numbers decreased in only a few patients after surgery.

Thus we were able to confirm experimental report on cells surviving in the circulation with high efficiency [11] and on the longevity of such cells [12] in patients [13]. These results are in contrast to the assumption that such circulating tumor cells are rapidly eliminated from the circulation, alluding mainly to a single publication in which the authors themselves conceded, that “the CTCs (circulating tumor cells) in dormancy candidates may have a different half-life than those shed from a primary tumor” and “in contrast to examining CTCs on slides, flow cytometry has a fluctuating background level of events in normal samples” [14]. Only ∼0.01% of such cells in the circulation may be able to generate metastatic foci [15].

In the present work we investigated the fraction of patients showing increasing CETC prior to the onset of adjuvant chemotherapy weeks after surgery and whether this is related to certain tumor properties and the therapeutic approach (breast-conserving surgery *versus* mastectomy).

Such cells might contribute to the metastasis formation observed in patients treated with surgery alone with a peak in recurrences 2–4 years after surgery and a later, more extended increase in metastatic disease between 6–15 years after removal of the primary tumor [16]. Surgery might, additionally, induce the growth of these remnant tumor cells by induction of, e.g., growth factors, a phenomenon that had previously been observed in experimental systems [17]. Disseminated tumor cells may, on the other hand, remain dormant [18,19] for certain times and be reactivated by the appropriate stimuli [20].

Today, however, only very few breast cancer patients are treated with surgery alone. The appraisal of breast cancer as a systemic disease led to the attempt to eliminate the remnant systemic tumor cells using chemotherapy. Indeed, this was highly successful and leads to considerably improved outcome of patients with breast cancer [21], even after 30 years of follow-up.

However, even with systemic chemotherapy, in some patients relapses occur, and we know that a considerable number of patients would have remained relapse-free without chemotherapy.

Therefore, we further investigated the fate of the CETC during systemic chemotherapy and showed that patients whose CETC did respond to systemic chemotherapy had a significantly better outcome than those whose tumor cells increased in spite of therapy [22]. The same is true for the behavior of the tumor cells during subsequent hormone blocking maintenance therapy [23].

Typically such curves in the behavior of the CETC are demonstrated during the course of the disease.

## 2. Materials and Methods

1 mL of anti-coagulated peripheral blood was obtained, according to ethics committee approval, and analyzed using the previously described microfluorimetric method, where assay method stability of the sample and reproducibility are extensively described [24].This volume was sufficient to detect cells in the pre-surgery as well as the post-surgery situation. In short, in order to compensate for shipping delays samples were subjected to red blood cell lysis at day 2 after blood drawing (usually with 95% viability) using 10 mL of erythrocyte lysis solution (Qiagen, Hilden, Germany) for 10 min in the cold, spun down at 700 g and rediluted in 1 mL of PBS. 10 μL of fluoresceinisothiocyanate (FITC)-conjugated mouse anti-human epithelial antibody (HEA) (Milteny, Bergisch Gladbach Germany) and 1 μL of phycoerythrin (PE)-labelled anti-CD45 were added to 100 μL of cell suspension, incubated for 15 min in the dark, readjusted to 1 ml and 20 μL of this suspension were used for measuring epithelial-antigen positive cells.

**Figure 1.**
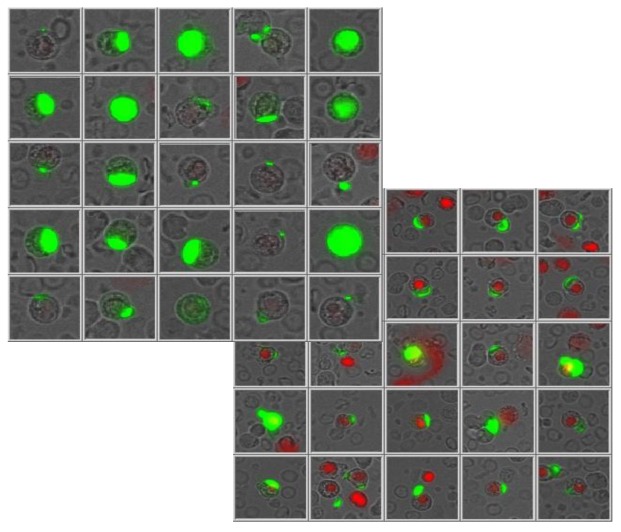
Gallery of vital (upper panel) and dead (lower panel) EpCAM-positive cells, typical cap formed surface staining covering a larger or smaller area on the cell. Dying cells have a permeable cell membrane allowing the nuclear stain propidium iodide to enter.

A defined volume of the cell suspension was applied to a defined area either on adhesion slides (Menzel Gläser, Braunschweig, Germany) or into wells of Elisa plates; the adherent cells were measured either using a Laser Scanning Cytometer (LSC^®^ Compucyte Corporation, Cambridge, MA, USA) and collecting the FITC-HEA and the PE-CD45 fluorescence using a photomultiplier (PMT) or using image analysis in the ScanR (Olympus, Munich, Germany). Values are displayed in scatter grams and histograms. Both approaches enable the user to locate cells contained within the positive population for visual examination and to take photos and fluoromicrographs. Figure 1, viability of the cells was visually detected and verified by Propidium Iodide (PI) staining (entering exclusively dying cells), looking for nuclear PI stain and exclusive surface EpCAM staining. Patients were followed for their CETC numbers from surgery to adjuvant chemotherapy, during adjuvant treatment, and some of them during the subsequent maintenance therapy. CETCs were analyzed at each visit, if possible at intervals of three months and later at more extended intervals. This allowed longitudinal follow-up of the CETCs. Patients were categorized according to their behavior into those with any increase, decrease or no change from surgery to the onset of chemotherapy, during chemotherapy and in some cases over a period of three years or if relapse occurred before this time. Statistical analyses were performed using the SPSS program, version 16.1.

## 3. Results

54 randomly selected patients who were operated on with breast conserving surgery (BCS) and 12 patients who received mastectomy during the same time were analyzed for their number of CETC suspected to be of tumor origin before surgery and before the onset of chemotherapy. Cell numbers before surgery ranged from undetectable to 47160 with a mean of 8008 and a median of 3876/mL for BCS and between 1000 and 17600/mL with a mean of 8562 and a median of 7224/mL for mastectomy. Thus there were no significant differences between the two patient groups with respect to pre-surgery CETC. Patient characteristics are given in Table 1 and Table 2, showing that T1 stages were more frequent in the BCS group as expected but N-stages, estrogen-receptor and HER/2neu status were comparable in both groups.

As we reported earlier, CETC numbers after surgery showed an initial increase in 80% of patients and subsequently could either further increase, decrease, or remain constant.

An increase until further treatment occurred in 20 patients, a decrease in 17 patients and no change in another 17 patients. Therefore, changes in the number of circulating tumor cells were roughly equally distributed between increase, decrease, and no change.

Increase or decrease from surgery to the onset of adjuvant chemotherapy was independent of tumor size but, surprisingly, significantly more increases occurred in N0 patients (15/30) *vs.* N1 patients (4/24) (p = 0.011), in hormone receptor-negative patients (7/10) *vs.* hormone receptor-positive patients (14/43) (p = 0.029), in HER2/neu− (15/29) *vs.* HER2/neu+ (3/17) (p = 0.022), and patients with initially low numbers (below 2500/mL) of circulating cells suspected to be of tumor origin (13/18) *vs.* initially high numbers (10/36) (p = 0.002). In contrast, no such differences were observed in patients scheduled for mastectomy. Of 12 patients treated with mastectomy, 4 had increasing, 5 decreasing and 3 no change in CETC.

So far only 5/54 (9.25%) patients with BCS have suffered relapse, but all 5 had increasing cell numbers after surgery.

The same is true for the 3/12 (25%) patients relapsing from the mastectomy group.

**Figure 2.**
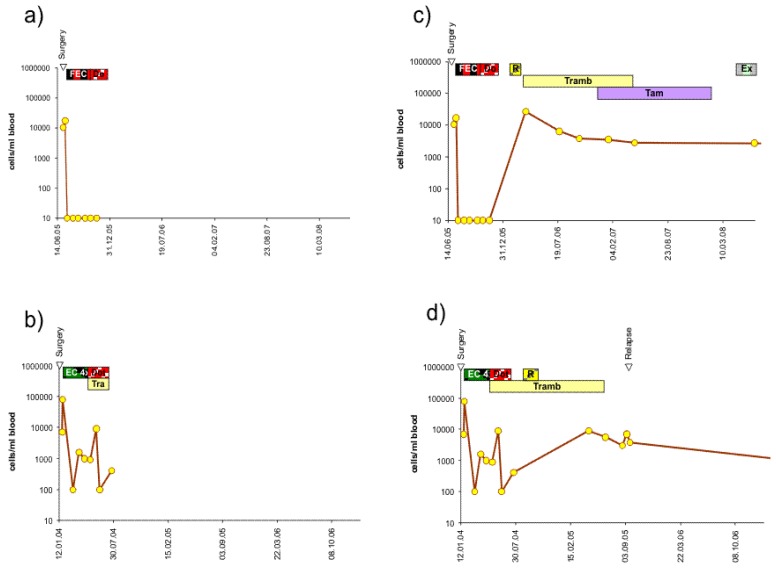
Typical courses of the behavior of vital cells during treatment in (a) and (b) an individual patient with estrogen receptor positive tumor in complete remission and (c) and (d) with relapse. Duration of treatment is shown as bars: FEC: 6 cycles of fluorouracil, epirubicin and cyclophosphamid; FEC Doc: 3 cycles of FEC and 3 cycles of docetaxel; R: local radiation to the breast; Tam: tamoxifen; Tramb: trastuzumab; Ex: exemestan.

**Figure 3.**
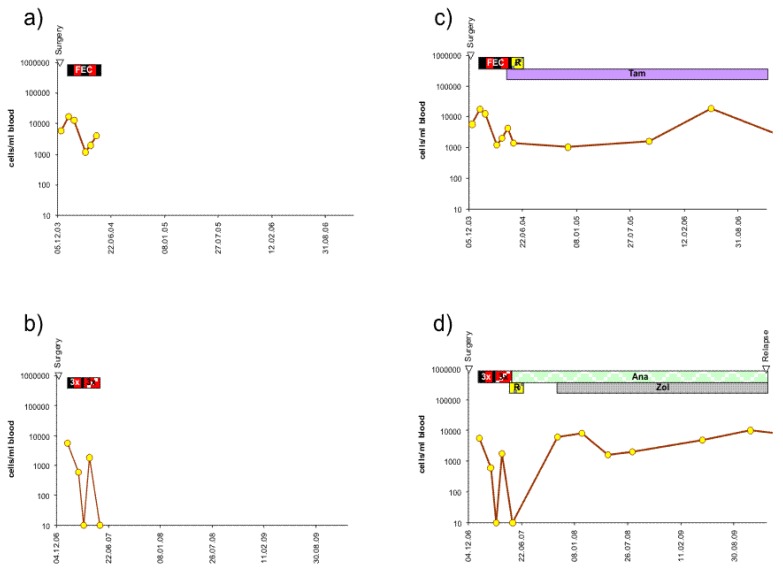
Typical courses of the behavior of vital cells during treatment in (a) and (b) an individual patient with HER2/neu positive tumor in complete remission and (c) and (d) with relapse. Treatment bars: as above. Ana: anastrozol; Zol: zoledronat.

**Figure 4.**
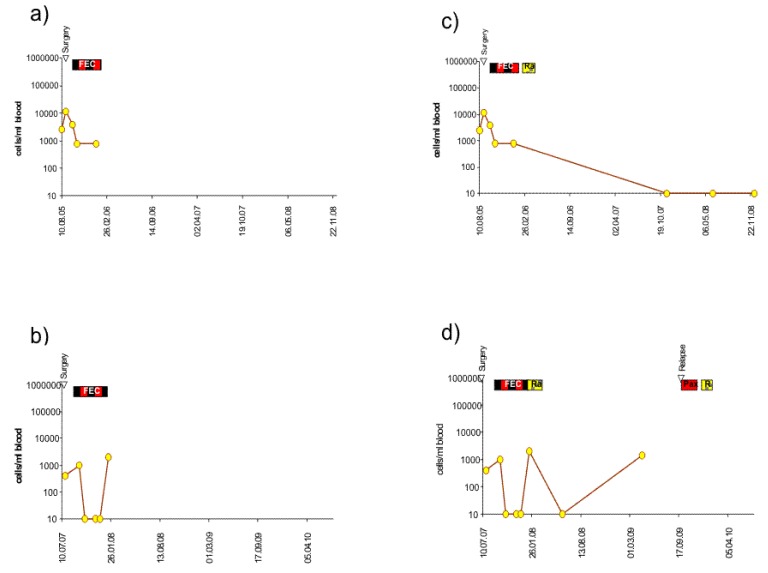
Typical courses of the behavior of vital cells during treatment in (a) and (b) an individual patient with triple negative tumor in complete remission and (c) and (d) with relapse. Treatment bars: as above. Pa: paclitaxel.

Almost all patients at our institution are treated with adjuvant therapy after surgery, most with adjuvant chemotherapy and/or antibody or hormone-blocking therapy according to their expression profile. This may delay or prevent relapse and lead to longer relapse-free or overall survival as compared to patients formerly treated with surgery only. We had previously shown that a re-increase of circulating epithelial tumor cells during adjuvant chemotherapy is an indicator of poor relapse-free survival. Typical courses of the number of circulating epithelial tumor cells during chemotherapy are shown in Figure 2a, 2b, Figure 3a, 3b and Figure 4a, 4b. With a maximum follow-up time of 7.7 years, all the relapses in the BCS group and the mastectomy group to date have occurred in the first three years. All 5 patients of the BCS group with these early recurrences also had increasing numbers of CETC during adjuvant chemotherapy, and this was also true for the 3 patients with mastectomy (Figures 2b, 3b and 4b).

Most patients are subsequently treated with hormone and/or antibody maintenance therapy. Typical courses of further development of the number of CETC is shown for examples from each of the three groups: HER2/neu positive, hormone receptor-positive and hormone receptor-negative in Figures 2c, 2d, 3c, 3d, 4c and 4d respectively. CETC were observed further increasing in patients from the HER2/neu positive, the hormone receptor-positive and the hormone receptor-negative patients eventually suffering from early relapse (Figures 2d, 3d and 4d).

We now have the first indications that even patients with decreasing CETC numbers during hormone-blocking maintenance therapy may, after cessation of hormone therapy, re-increase in cell numbers and suffer relapse, possibly the group of late relapses.

## 4. Discussion

In breast cancer early relapses are still a problem even if metastases can occur up to 20 years after primary diagnosis. It is assumed that tumor cells can be shed from the tumor from the time when the tumor starts to become vascularized, which occurs most probably starting from the 1 million cell size, which would correspond to approximately 1 mm in diameter. At that point, the tumor is not yet detectable. Released cells might reside dormant in some occult loci until reactivated to grow into metastases [25]. The early peak of relapses in patients treated with surgery only, at about 24 months after surgery, led Retsky [26] to propose a model where such dormant cells are reactivated by stimuli produced after surgery, e.g., due to wound healing, and to propose a new approach to treat newly diagnosed breast cancer [27].

We herein add additional evidence as to how surgery might influence the outcome in primary breast cancer patients, but also probably in other malignant diseases. In analogy to earlier observations [5,6] we reported [9,28] and here confirm in a larger group of patients, that surgery can lead to release of epithelial cells, part of which may be cells from the cancer. Increased numbers until the onset of the next therapeutic action would indicate that cells that were released during surgery remain in the circulation until e.g. chemotherapy, whereas no change would indicate that, during removal of the main tumor load, no further cells are seeded into circulation, and a decrease would even indicate that after removal of the seeding source, cells in the circulation are eliminated. All three instances were found to occur.

The observation that increasing numbers of epithelial cells were found preferentially in patients with N0 tumors indicates that in these patients fewer cells may have been released during tumor growth, possibly due to low vascularization [29], and surgery had a higher contribution to cell release. The same may be true in patients with hormone receptor-negative and HER2/neu-negative tumors, and is substantiated by the finding that patients with initially low numbers of CETC more often had increasing cell numbers after surgery. Thus, surgery may contribute to metastasization by sending an additional wave of tumor cells into the body. In spite of subsequent chemotherapy and maintenance therapy all of the relapsing patients in our patient group had shown an increase in cell numbers due to surgery. Thus, even if not all patients in whom cells were mobilized due to surgery suffered relapse, those with more aggressive cells, surviving subsequent treatments may be at higher risk of relapse. We hypothesize that during surgery cells are forced into the lymphatics and are subsequently drained into blood. Lymph nodes may, therefore, not be an effective barrier [10]. One of the most recent propositions may be the omission of axillary dissection if the sentinel lymph node is only minimally involved, but this is heavily debated [30,31].

Most patients, nowadays, are treated with adjuvant therapy subsequent to surgery. Although CETC were effectively reduced by adjuvant therapy, in patients with early relapses, rapid re-increases occurred even during chemotherapy (Figures 2b, 3b and 4b). We can, to a certain extent, try to calculate the influence of chemotherapy.

In a patient with a rapidly growing tumor, the doubling time of the tumor cells might be assumed to be 36 days. It takes about 20 doublings, which is two years, for her tumor to grow to a still undetectable size of 1 mm and a total of three years for her tumor to grow to a size of 10 mm. It may be detected at that time and removed by surgery. At two years of growth her tumor had reached 10^6^ cells, vascularization is assumed to be initiated at that time and cells can start to leave the primary tumor. If a metastasis starts to grow from the time of vascularization at the same rate as the primary tumor, it will be detectable around two years after surgery even without the assumption of dormancy.

The cells in the metastasis grow to 1000 cells during the year from vascularization until surgery of the primary tumor. If, however, as in most breast cancer patients, the patient is treated with adjuvant chemotherapy, and, due to rapid growth, her cells are very sensitive to chemotherapy, the number of cells in her metastasis might be assumed to be reduced to 1, which is 10 doubling times corresponding to a delay of about one year.

If this remnant cell restarts re-growing at the same rate as before, it will take about 30 doublings or about three years from surgery for the metastases to become measurable [32].

The same is true if cells are seeded from the tumor only at surgery; this would postpone the detection of metastases for only one year, as compared to early seeding.

The results of these calculations correspond to our results that all of the early relapses occurred during the first three years after surgery and are in agreement with the early recurrence peak [33].

In contrast, a patient with a malignant lump of 10 mm size detected in her breast often tells the physician: “Oh this lump has been in my breast for years and years and was always considered to be benign”. This may be a slowly growing tumor with a cell doubling time of about 120 days which implies that to grow from one tumor cell to 1000 tumor cells, meaning 10 doublings, has taken about three years. It takes another three years for this tumor to grow to 10^6^ cells, a size of 1 mm, which is still not detectable by the currently available imaging systems. Still three more years pass until the tumor reaches the size of 10^9^ cells equal to a tumor of 10 mm size, when it is detectable either by imaging or by the patient herself. The tumor has then been growing for nine years before it is detected and most probably is removed surgically.

Metastases developing from this tumor can have started about three years before the tumor is detected and removed. If the metastases grow at the same rate as the tumor, a metastasis can be expected to be detected the earliest at a size of 10 mm, which it reaches six years after the primary tumor has been removed. This would be the case if the patient has had no other treatment but surgery. However, the patient will also be treated with chemotherapy, which, due to slow growth, may have little impact on the cells in circulation (Figures 2a, 3a and 4a) as well as in the metastasis. Most probably her tumor is estrogen receptor-positive and she is subsequently treated with hormone receptor modulating therapy [34]. Tamoxifen silences circulating tumor cells [35], leading to a slow reduction probably by senescence but rarely completely eliminates the CETC (Figures 2c and 3c), especially not the cells in the metastases. Indeed, we have recently observed late occurrence of metastases after tamoxifen treatment has ceased. Thus hormone receptor modulation treatment may postpone metastasis formation compatible with a later more sustained distribution between six and 15 years.

## 5. Conclusions

This indicates that the “age” and thus the growth behavior of the primary tumor may be extremely important for the treatment of the individual patient, but so far it is not possible to determine from tumor size how long a cancer has been growing.

In this situation the behavior of the CETC and the speed of reappearance after chemotherapy might provide a tool to determine the growth activity of the tumor. This may allow timely therapeutic interventions such as anti-angiogenetic approaches, but probably in addition to treatments as proposed by Epstein [36].

## Figures and Tables

**Table 1.** Tumor characteristics of patients with increasing, decreasing and no change in CETC after treatment with breast conserving surgery.

**Breast-conservering therapy patients**				**Total 54**
	**increase**	**decrease**	**no change**	**not analyzed**
tumor stage				
T1	13	10	13	
T2	5	6	5	
T > 2	0	1	0	
T?	1			
lymphnodes				
N0	15	8	7	
N1	4	9	11	
hormone receptor				
HR+	14	15	14	
HR−	7	1	3	
HER2/neu				
HER2/neu+	3	9	5	8
HER2/neu−	15	5	9	
CETC before surgery				
high(>2000)	10	13	13	
low(<2000)	13	2	3	

**Table 2.** Tumor characteristics of patients with increasing, decreasing and no change in CETC after treatment with mastectomy.

**Mastectomy patients**				**Total 12**
	**increase**	**decrease**	**no change**	**not analyzed**
tumor stage				
T1	1	1	0	
T2	2	3	2	
T > 2	1	1	1	
lymphnodes				
N0	2	2	2	
N1	2	3	1	
hormone receptor				
HR+	3	5	2	1
HR−	1	0	0	
HER2/neu				
Her2+	2	0	1	1
Her2−	2	5	1	
CETC before surgery				
high(>2500)	4	4	1	
low(<2500)	1	2	0	
